# Freeze-dried noncoagulating platelet-derived factor concentrate is a safe and effective treatment for early knee osteoarthritis

**DOI:** 10.1007/s00167-023-07414-y

**Published:** 2023-06-28

**Authors:** Tadahiko Ohtsuru, Masaki Otsuji, Jun Nakanishi, Norimasa Nakamura, Stephen Lyman, Hiroto Hanai, Kazunori Shimomura, Wataru Ando

**Affiliations:** 1 Omiya Knee Osteoarthritis Clinic, Saitama, Japan; 2 Department of Orthopedic Surgery, Tokyo Women’s Medical University School of Medicine, Tokyo, Japan; 3 Yokohama Knee Osteoarthritis Clinic, Kanagawa, Japan; 4 Tokyo Knee Osteoarthritis Clinic, Tokyo, Japan; 5 Osaka Health Sciences University, Osaka, Japan; 6 Hospital for Special Surgery, New York, NY USA; 7 Kyushu University School of Medicine, Fukuoka, Japan; 8 Department of Orthopedic Surgery, Osaka University Graduate School of Medicine, Osaka, Japan

**Keywords:** Osteoarthritis, Platelet-rich plasma, Freeze-dried, Clinical outcomes, Knee osteoarthritis

## Abstract

**Purpose:**

While a wide variety of platelet-rich plasma (PRP) solutions has been developed, innovation continues. In this case, the freeze-dried platelet factor concentrate (PFC-FD) represents another step in PRP refinement. The preparation of PFC-FD at a central laboratory with freeze drying for shelf stabilization should provide additional quality improvements if clinical effectiveness can be demonstrated. Therefore, this study was undertaken to assess the safety and effectiveness of PFC-FD in a prospective open-label trial of patients suffering from knee osteoarthritis (OA).

**Methods:**

312 consecutive knee OA patients (67% female, mean age 63 ± 10 years), were prospectively recruited in an outpatient knee clinic in Japan. Of these, 10 (3.2%) were lost to follow-up at < 12 months and 17 (5.5%) sought additional knee therapy during the follow-up period. The primary outcome of interest was achievement of the OMERACT-OARSI responder criteria with secondary outcomes of adverse events and PROMs scores 1, 3, 6, 12 months following a single PFC-FD injection.

**Results:**

285 patients (91%) completed 12 month PROMs. The 17 who sought additional therapy were considered failures leaving an effective sample size of 302 for our primary outcome in which 62% of patients achieved OMERACT-OARSI responder status by 12 months. This varied by OA class with Kellgren–Lawrence grade 4 patients 3.6 times less likely to be responders than grade 1–2 patients. 6% of patients experienced a non-serious adverse event, primarily pain or swelling at the injection site.

**Conclusions:**

PFC-FD provides an observable clinical improvement in 62% of knee OA patients at 12 months post-injection with very little risk of any clinically relevant adverse event. Of course, nearly 40% of patients did not experience an observable clinical improvement, primarily among those with worse KL grades.

**Level of evidence:**

Therapeutic, Level II.

**Supplementary Information:**

The online version contains supplementary material available at 10.1007/s00167-023-07414-y.

## Introduction

Management of painful knee osteoarthritis (OA) remains a challenging dilemma for clinicians and patients alike. Established conservative therapies address symptoms while lacking disease modifying properties. The search for a truly disease modifying OA treatment has lead physicians and scientists to biologic therapies such as mesenchymal stem cell or platelet-rich plasma (PRP) injections, the latter of which has become a common treatment option. An extension of PRP is the use of noncoagulating platelet-derived factor concentrate (PFC) [1], which maximizes platelet concentration while removing fibrinogen.

To stabilize the PFC, the solution is freeze-dried (PFC-FD) at a central laboratory. This allows the PFC-FD to be stored at room temperature for up to 12 months prior to use in patients, which allows for more flexibility clinically. Additionally, the central laboratory should theoretically provide more consistent quality of solution preparation than an individual clinic’s PRP processing methods.

While PFC-FD may offer several theoretical processing advantages to improve clinical workflow, there are currently no published clinical outcomes evaluations of PFC-FD. Therefore, this study was undertaken as a single-arm open-label trial of PFC-FD of patients presenting at an outpatient knee clinic with knee OA Kellgren–Lawrence (KL) grade 1 or higher. The study aims were to assess PFC-FD for: (1) evidence of clinical responsiveness based on Outcome Measures in Rheumatology-Osteoarthritis Research Society International (OMERACT-OARSI) responder criteria [8], (2) safety based on adverse event frequency, and (3) effectiveness based on patient reported outcome measures of pain and function.

## Methods

Consecutive knee OA patients (*N* = 312) presenting at an outpatient knee clinic in Tokyo, Japan, were prospectively enrolled from October 2018 to April 2020 of which 285 completed full follow-up. Patients were eligible for enrollment if they agreed to undergo PFC-FD monotherapy, had no history of prior knee surgery, had no prior knee injection within 1 month, were not currently taking antithrombotic or antiplatelet medications, and had no known or suspected autoimmune disease, chronic infectious disease (e.g. Hepatitis B or C, HIV), or coagulopathy that might increase risk of an adverse reaction to biologic therapy. Patients wishing to undergo combined PFC-FD therapy with hyaluronic acid or stem cell injections were excluded. Eligible patients were informed that they were receiving an experimental treatment and were consenting to its administration. This study adhered to the Declaration of Helsinki guiding human subjects research, was conducted under the standards required by the Japanese Regenerative Medicine Promoting Act on the Safety of Regenerative Medicine and was approved by the ethics committee of Katsujukai Medical Corporation (#0002).

### Baseline assessment

Enrolled patients underwent a baseline assessment consisting of radiographs or magnetic resonance imaging (MRI) of the knee to confirm the diagnosis and severity of knee OA using the KL grading system. Patients also completed a battery of patient reported outcome measures (PROMs) at this time. Specifically, they completed the previously validated Japanese language version of the knee osteoarthritis and injury outcomes score (J-KOOS) [7] and a pain visual analogue scale (VAS), which measured pain at rest.

### Injection preparation

To process PFC-FD, 40 ml of whole blood was drawn from the patient and sent to a central cell culture processing laboratory (CellSource, Tokyo, Japan). The blood was centrifuged for 280×*g* and rested for 10 min at room temperature. Top layer plasma was collected, centrifuged for 1,400xg, and again rested at room temperature for 10 min before the precipitated PRP was collected. To compose the platelet-derived factor concentrate (PFC), 5.0 ml of phosphate buffered saline and 1.5 ml of 2% CaCl_2_ was added to the PRP and suspended for 20 min at room temperature. The solution was then passed through a 0.45 µm filter to remove cell components before being freeze-dried. Prior intraarticular injection, the PFC-FD solution was mixed with 6 ml of sterile saline.

The concentrations of cytokines and growth factors present in PFC-FD were assessed using enzyme-linked immunosorbent assay (ELISA) on PFC-FD drawn from the knees of three healthy volunteers. Quantikine ELISA kits (R&D systems, Minneapolis, MN, USA) were used for the measurement of the concentrations for selected growth factors and cytokines: PDGF-BB (DBB00), TGF-β(DB100C), VEGF (DVE00), EGF (DEG00) and IL-1ra (DRA00B). All procedures were conducted per manufacturer’s instructions. Observed values are reported in Table 1.

**Table 1:** ELISA growth factor and cytokine concentrations from the PFC-FD from 3 healthy volunteers

Growth factor/cytokine	Mean (pg/mL)	s.d (pg/mL)
Platelet derived growth factor BB (PDGF-BB)	216	82
Transforming growth factor β (TGB-β)	5498	810
Vascular endothelial growth factor (VEGF)	37	17
Epidermal growth factor (EGF)	35	5
Interleukin 1 receptor antagonist (IL-1ra)	182	80

### Outcomes

Follow-up consisted of repeated PROMs (J-KOOS and Pain-VAS) at 1, 3, 6 and 12 months post-PFC-FD injection. Outcomes considered were the J-KOOS5 (averaged score of all 5 J-KOOS domain scores), 5 domains of the J-KOOS, the Pain-VAS, and OMERACT-OARSI responder criteria for these PROMs. Further, safety was assessed by thorough chart review for both serious and mild adverse events at the end of the follow-up period. A total of 10 patients were lost to follow-up (3.2%) while 17 (5.5%) sought additional treatment during the follow-up period. Interim follow-up data for these patients were retained.

We assessed the change in PROMs scores in a variety of ways. First, we considered the effect size with the interpretation that an ES effect size greater than 0.8 was considered a large effect, 0.5–0.8 was a moderate effect, and 0.2–0.5 was a small effect. We further assessed the improvement or change in PROMs based on a 10 mm decrease in pain-VAS and a 10-point improvement for all KOOS domains based on recommendations from the KOOS developer [11]. This minimally clinically important change (MCIC) is a useful tool for determining whether the change in score exceeds a threshold for which the patients can sense a change in their state of knee health for that domain. We were unable to find an anchor-based estimate of the MCIC for the KOOS-5 for patients with knee OA in the literature so we relied on the developer’s recommended 10-point improvement as the MCIC [11].

### Statistical analysis

An a priori power analysis determined that we would need a minimum of 263 patients to achieve 90% power to detect a responder rate of 60% as we considered the possibility of a placebo effect of up to 50% simply due to knee injection [2]. We anticipated up to 15% patient loss to follow-up so increased our recruitment target to 305 patients. A total of 312 were ultimately recruited.

Our primary study outcome was achievement of the OMERACT-OARSI responder criteria with secondary outcomes of KOOS-5 scores and safety evaluation. All other PROMs (KOOS domains, Pain-VAS) were considered tertiary outcomes. The 17 patients who sought additional treatments were classified as non-responders once they sought additional treatment while the 10 lost to follow-up were excluded from the responder analysis once they were lost.

Descriptive statistical analysis consisted of means and standard deviations or medians and intraquartile range for continuous variables depending on the normality of the underlying distributions. Wilks–Shapiro tests were used to evaluate normality. For discrete variables, frequency counts and percentages were implemented. Univariate analysis was performed using repeated measures analysis of variance for PROM outcomes. A sub-analysis was also performed by KL grade due to concerns over difference in response by OA severity over time. OMERACT-OARSI responder criteria [8] were assessed by Chi-square analysis.

Multivariable logistic regression analysis was performed using a binary responder criteria indicator as the outcome. This model was adjusted for age, sex, body mass index, and KL grade. All analyses were performed using IBM SPSS Statistics version 27 (Armonk, NY, USA). A *p* value of < 0.05 was considered statistically significant.

## Results

A total of 312 consecutive patients were successfully enrolled and underwent PFC-FD injection administered by their treating surgeon. All injections were to a single knee only. Patients averaged 63.4 years of age (min 30, max 88), were 67.7% female, and a plurality (44.4%) had KL grade III OA (Table 2). No sex differences were observed so these results are not reported. For all subsequent analyses KL grade I and II were combined, because only 5% of the cohort were KL grade I. Ten patients (3.2%) were lost to follow-up prior to 12 months and 17 (5.5%) were censored once they sought additional treatments. A total of 87% of requested PROMs were returned including 100% at both baseline and 97% at 12 months.

**Table 2:** Baseline patient characteristics

	Number	Percent (%)	Mean SD	Range
All	312			
Age			63.5 ± 10.0	30–88
≤ 59	104	33		
60 ≤ 69	129	41		
70 ≤	79	25		
Sex				
Male	102	33		
Female	210	67		
BMI			24.2 ± 4.4	15.6–46.3
< 25.0	201	64		
25.0 ≤	111	36		
KL grade				
KL-1	17	5		
KL-2	96	31		
KL-3	138	44		
KL-4	61	20		

### PROMs

**Fig. 1:**
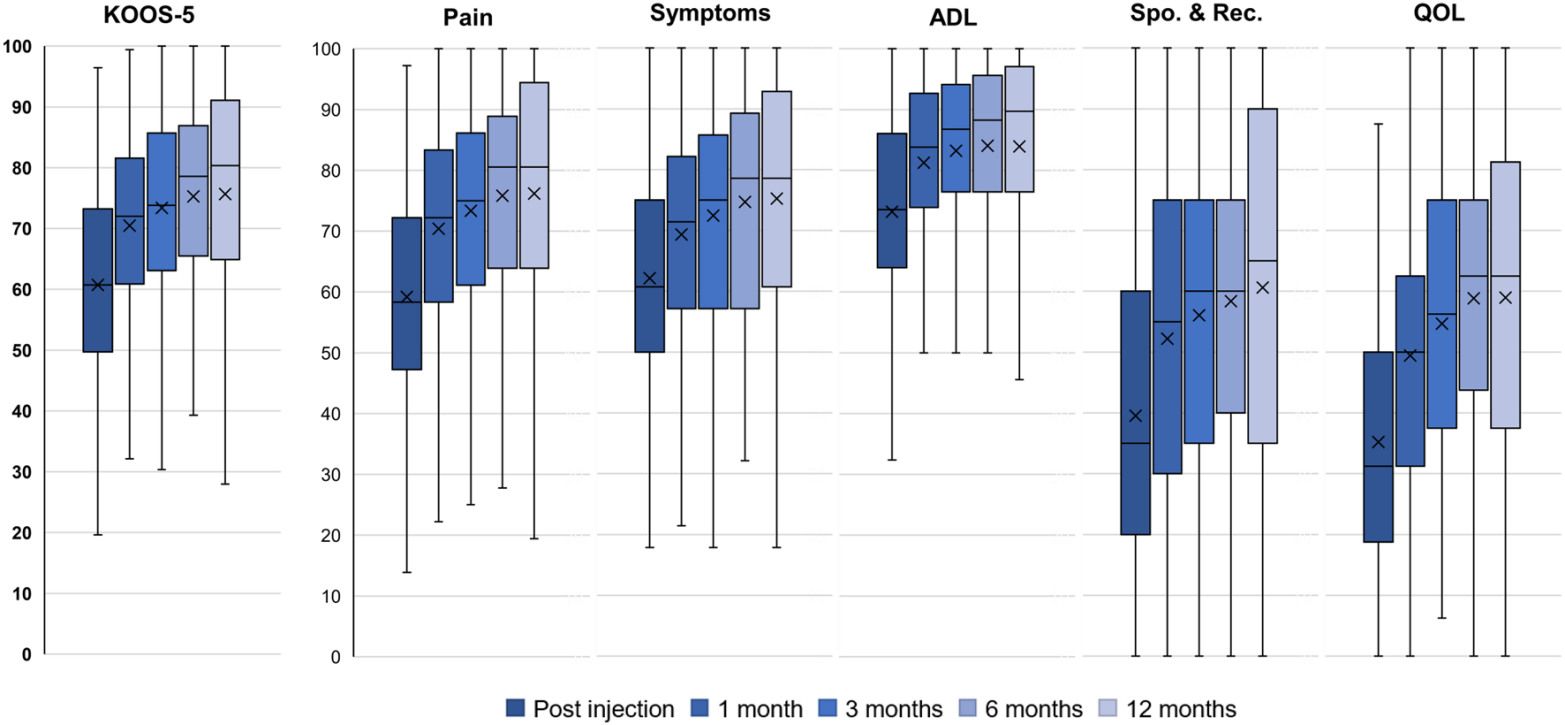
Knee Injury and Osteoarthritis Outcome Score at baseline and 1, 3, 6, 12 months follow-up

For the overall cohort, all PROMs domains improved significantly (*p* < 0.01) at all timepoints compared to baseline PROM scores. Average pain-VAS scores achieved both a large effect size (0.91) and MCIC (≥ 10 mm) by 1 month and maintained these improvements through 12 months while KOOS scores were more nuanced in their response to PFC-FD injection (Fig. 1). The KOOS-5 and all KOOS domain scores improved to at least a moderate effect size by 3 months post-PFC-FD injection (ES = 0.52–0.78). Only the KOO-5 (0.81), Pain (0.85), and QOL (0.94) domains achieved a large effect size by 6 months, which was maintained through month 12 while all other domains maintained moderate effect sizes throughout the post-injection period.

All domains also reached the a priori MCIC of 10 points by 3 months post-injection and remained above this threshold out to 12 months. However, the overall improvement varied widely by domain. For example, KOOS-QOL improved by over 23 points by 6 months, but KOOS-ADL never improved beyond 12.2 points.

When considered by OA grade, KL grades 1–3 followed a similar pattern to that seen overall with large improvements achieved by 1–3 months post-injection. However, for KL 4 patients, no domain achieved a large effect size at any timepoint and an MCIC of 10 points was only achieved at 1 month and maintained out to 12 months for the KOOS-Pain, QOL, and sports and recreation domains.

**Fig. 2:**
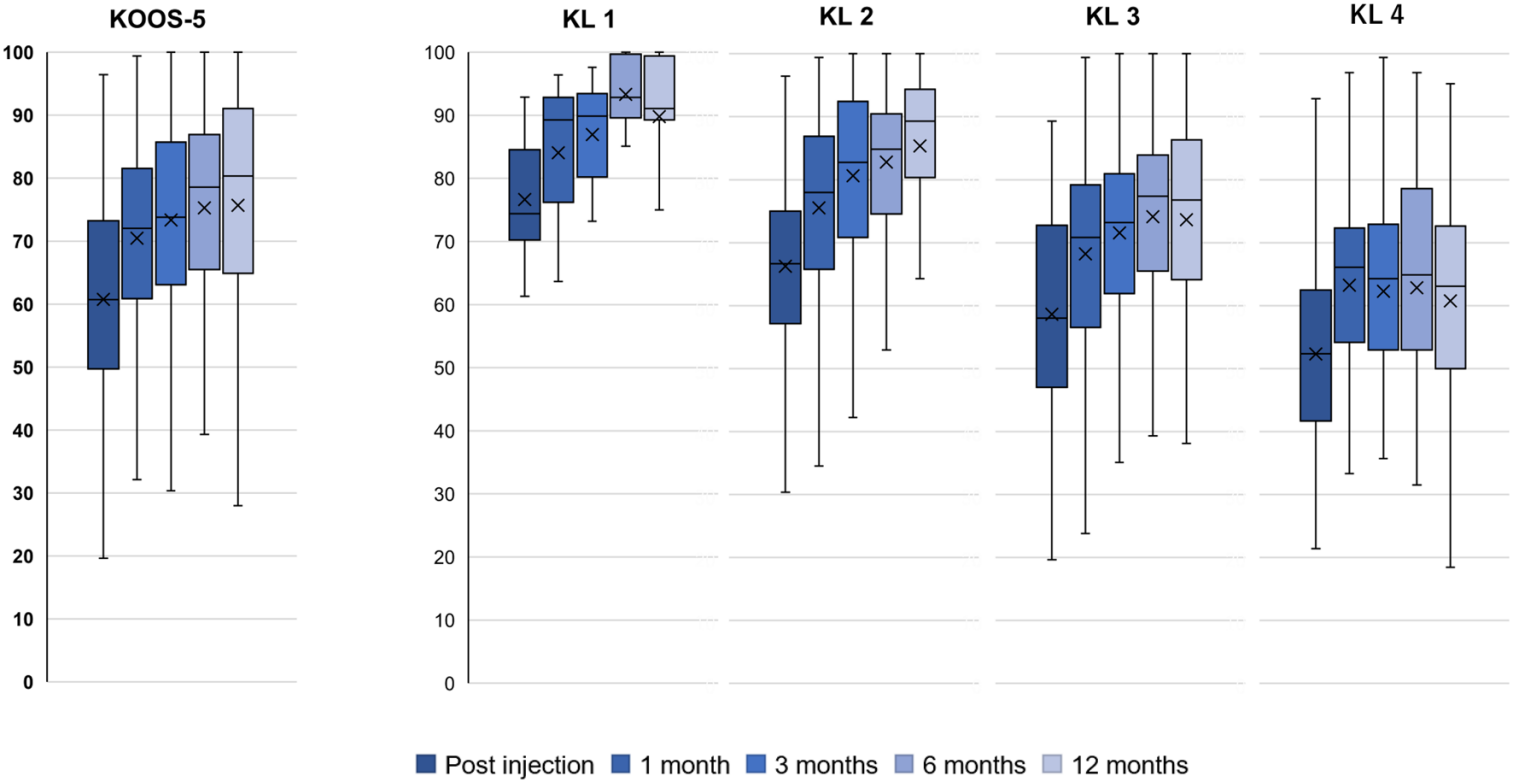
KOOS Score change by grade of knee osteoarthritis (KOOS-5)

The patterns of improvement also differ by KL grade (Fig. 2 for KOOS-5, other KOOS sub-domain figures available in Appendix). KL grade 1–2 patients demonstrated large improvements by 1 month and these improvements continued to increase in magnitude out to 12 months for all PROMs scores. Grade 3 patients followed a similar pattern, but plateau at 6 months. Grade 4 patients plateaued at 1 month with only extremely small improvements beyond that timepoint and only in the QOL and sports and recreation domains. Other PROMs in Grade 4 patients declined after 1 month. Multivariable regression revealed that KL grade significantly modified change in PROMs scores for KOOS-5, KOOS-Pain, KOOS-QOL, and KOOS-Sports and Recreation.

### Responder criteria

**Fig. 3:**
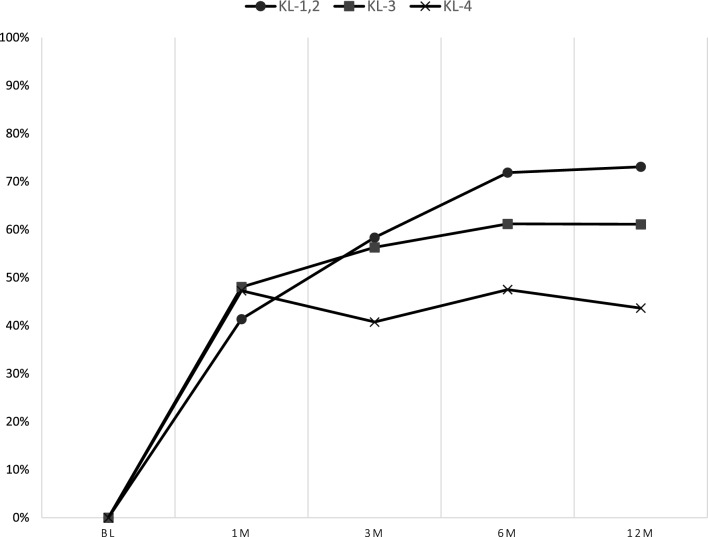
Change of responder rate by grade of knee osteoarthritis

A majority of patients (54%) were considered responders by the OMERACT-OARSI criteria by 3 months and 59% were responders by 12 months (Table 3). This varied widely by OA grade (Fig. 3). Nearly 70% of KL grade 1–2 patients were considered responders by 12 months, while just 41% of grade 4 patients were responders by this timepoint.

**Table 3:** Responder Criteria Achievement by timepoint and patient characteristic

Post injection	1 month	3 months	6 months	12 months
Variable	% Responder	% Responder	% Responder	% Responder
All	45 (128/276)	54 (142/259)	61 (113/184)	59 (177/302)
Age				
≤ 59	43 (40/94)	56 (50/89)	63 (36/57)	59 (61/103)
60 ≤ 69	51 (57/112)	53 (54/101)	64 (46/72)	63 (78/123)
70 ≤	44 (31/70)	55 (38/69)	56 (31/55)	50 (38/76)
Sex				
Male	51 (43/84)	57 (44/77)	53 (26/49)	55 (53/97)
Female	44 (85/192)	54 (98/182)	64 (87/135)	60 (124/205)
BMI				
< 25.0	46 (83/181)	52 (89/170)	62 (77/124)	61 (118/195)
25.0 ≤	47 (45/95)	60 (53/89)	60 (36/60)	55 (59/107)
KL grade				
KL-1	44 (7/16)	53 (8/15)	80 (8/10)	56 (9/16)
KL-2	42 (35/83)	62 (47/76)	71 (36/51)	71 (67/94)
KL-3	48 (60/125)	56 (66/117)	60 (51/85)	57 (77/134)
KL-4	50 (26/52)	41 (21/51)	47 (18/38)	41 (24/58)

Among the 17 patients who were considered treatment failures, 3 sought surgical intervention (2 total knee arthroplasty, 1 meniscectomy), 6 sought additional injections, and 8 transferred to another clinic or hospital for follow-up care with additional treatments unknown, but assumed to have occurred.

Regression analysis revealed that only KL Grade was a risk factor for non-responder status (Table 4). KL Grade 3 patients were 1.8 times more likely to be a non-responder than Grade 1–2 patients while Grade 4 patients were 3.6 times more likely to be non-responders.

**Table 4:** Multivariable regression to identify risk factors for non-responder status

Variable	OR	95% CI	*p* value
Age	1.01	0.98–1.04	0.503
Male Sex	1.56	0.90–2.70	0.113
BMI	0.99	0.94–1.05	0.822
Kellgren–Lawrence Grade			
1 & 2	Reference	Reference	–
3	1.83	1.01–3.32	0.047
4	3.63	1.73–7.61	0.001

### Adverse events

There were no serious adverse events following injection (Table 5). Just 17 patients (6%) reported mild adverse events with the most common complaint being pain and/or swelling at the injection site (16 patients). One additional patient reported a fever above 37° Celsius, which resolved without treatment within 48 h. There were no injection site infections reported in this patient cohort.

**Table 5:** Adverse events following PFC-FD injection

Type of adverse event	(%)
Serious adverse events	0 (0)
Non-serious adverse events	17 (5)
Treated site swelling	2 (< 1)
Treated site pain	3 (1)
Treated site swelling with pain	11 (4)
Fever	1 (< 1)

## Discussion

The main finding of this single-arm open-label trial of a monotherapy PFC-FD injection for knee OA is that this treatment is both safe and effective in certain patients. The treatment appears most effective in patients with early OA with moderate effectiveness in KL grade 3 patients.

Our understanding of the mechanisms by which PRP injections may be acting on human cartilage is rapidly evolving. Originally, cell concentrations were believed to be most important in achieving a clinical response. However, recent reports suggest the activity of exosomes, cytokines, and other growth factors may play a larger role than previously understood [4, 6, 12].

Regardless of how these injections are acting biologically, a large meta-analysis of over 1400 PRP injections across 34 RCTs has demonstrated a consistent advantage in outcomes for PRP versus placebo and some evidence of an advantage of PRP injections over hyaluronic acid or steroid injections [3]. In fact, across the 34 studies, in no instance was PRP found to be inferior (even non-significantly) than the alternative treatment option. This provides clear evidence that PRP injections provide some clinical benefit in the setting of knee OA.

Previous evaluations of PRP effectiveness in similar cohorts (Japanese patients suffering from symptomatic knee OA) have been limited by small sample sizes, short follow-up duration, and variable PRP preparation methodologies [5, 12–15]. Previous studies have reported OMERACT-OARSI responder achievement of 58–78% of injected knees at between 1 and 24 months [5, 10]. Like our own findings, none of these prior studies reported any injection site infections.

The most similar project to our own used LR-PRP in 260 patients with outcome followed out to 24 months [4]. Kenmochi, et al., reported 12-month responder achievement of 78% and 24 months of 77% [4]. However, these patients received a minimum of 4 PRP injections 4 weeks apart (mean 5.8 injections) and 13% of their cohort was KL Grade I. In our own study we observed 59% response achievement by 12 months with a single PFC-FD injection with just 5% of our cohort comprised of KL Grade I patients.

While outcomes appear similar between PFC-FD and other PRP injections, two distinct advantages of PFC-FD should be considered. First, the shelf stable room temperature storage of PFC-FD provides clear supply chain efficiencies that cannot hoped to be achieved with prior PRP preparation methods. Second, the uniform preparation methods should theoretically provide a more stable and consistent product than PRP products created by clinical teams of varying levels of experience on equipment of various manufacturing origin. While there may be concerns over the potential for contamination due to additional processing steps involving sample transport, these good clinical practice approved laboratories have strict control processes in place as well as extensive documentation requirements, which far exceed the expected quality controls with locally processed samples. Further, there have been no reports of suspect contamination due to central processing of this material.

This study suffers from several limitations, particularly that the patients were not randomized to PFC-FD or an alternative conservative treatment for knee OA such as another PRP injection or hyaluronic acid injection. Further, there is no comparison group as every patient was treated with the same regimen. This is particularly relevant, because there is a well-known placebo effect for knee injections even absent the presence of active biological materials [9]. Additionally, the time permitted between previous injections and enrollment was relatively short (1 month) since this study was undertaken at a private clinic not linked with the national healthcare system. While these criticisms are warranted, the study was conducted under the Japanese regulatory body governing regenerative medicine, an organization that has been hesitant to approve randomized study designs. Nevertheless, the study was prospective with rigorously applied inclusion criteria and an overall follow-up rate of 97%. We hope that these limitations can be used to encourage the Japanese authorities to allow future investigations, which include a comparison group for head-to-head evaluation of this PFC-FD therapy.

## Conclusion

This study has demonstrated that PFC-FD injection for knee OA provides a clinical response and is both and effective, particularly in patients with early to moderate knee OA (KL grades 1–3). While the disease modifying nature of this injection remains unknown, this study provides evidence for its safe and effective use as another tool in the conservative management toolbox of physicians treating patients with knee OA. Further, it remains unknown whether the PFC-FD injection can be used clinically for other knee pathology.

### Supplementary Information

Below is the link to the electronic supplementary material.

Supplementary file1 (PDF 117 KB)

Supplementary file2 (PDF 121 KB)

Supplementary file3 (PDF 112 KB)

Supplementary file4 (PDF 127 KB)

Supplementary file5 (PDF 126 KB)

Supplementary file6 (DOCX 60 KB)

## Data Availability

Data collected for this study was prospectively generated and largely used for clinical care. An appropriately de-identified dataset is available upon reasonable request.
