# Supplementary figures and images for: Freeze-dried noncoagulating platelet-derived factor concentrate is a safe and effective treatment for early knee osteoarthritis
Source: Knee Surg Sports Traumatol Arthrosc. 2023 Jun 28;31(11):4716–23. doi: 10.1007/s00167-023-07414-y (PMC10598078; doi:10.1007/s00167-023-07414-y)

Supplementary Figure 1. KOOS Score change by grade of Knee Osteoarthritis. (Pain)

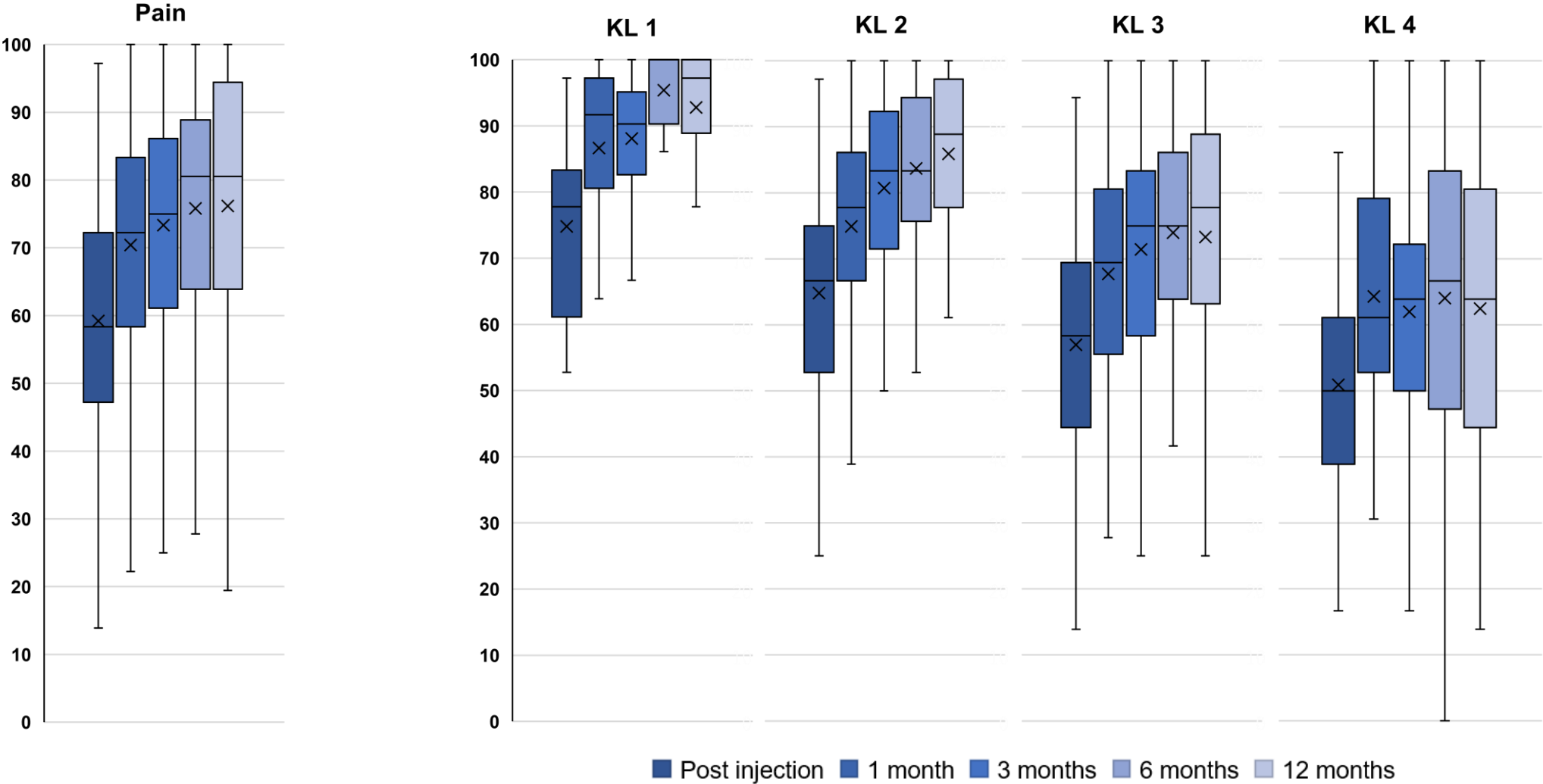

Supplement: Supplementary file 1 — Supplementary file1 (PDF 117 KB) [file 167_2023_7414_MOESM1_ESM.pdf]

Supplementary Figure 2. KOOS Score change by grade of Knee Osteoarthritis. (Symptoms)

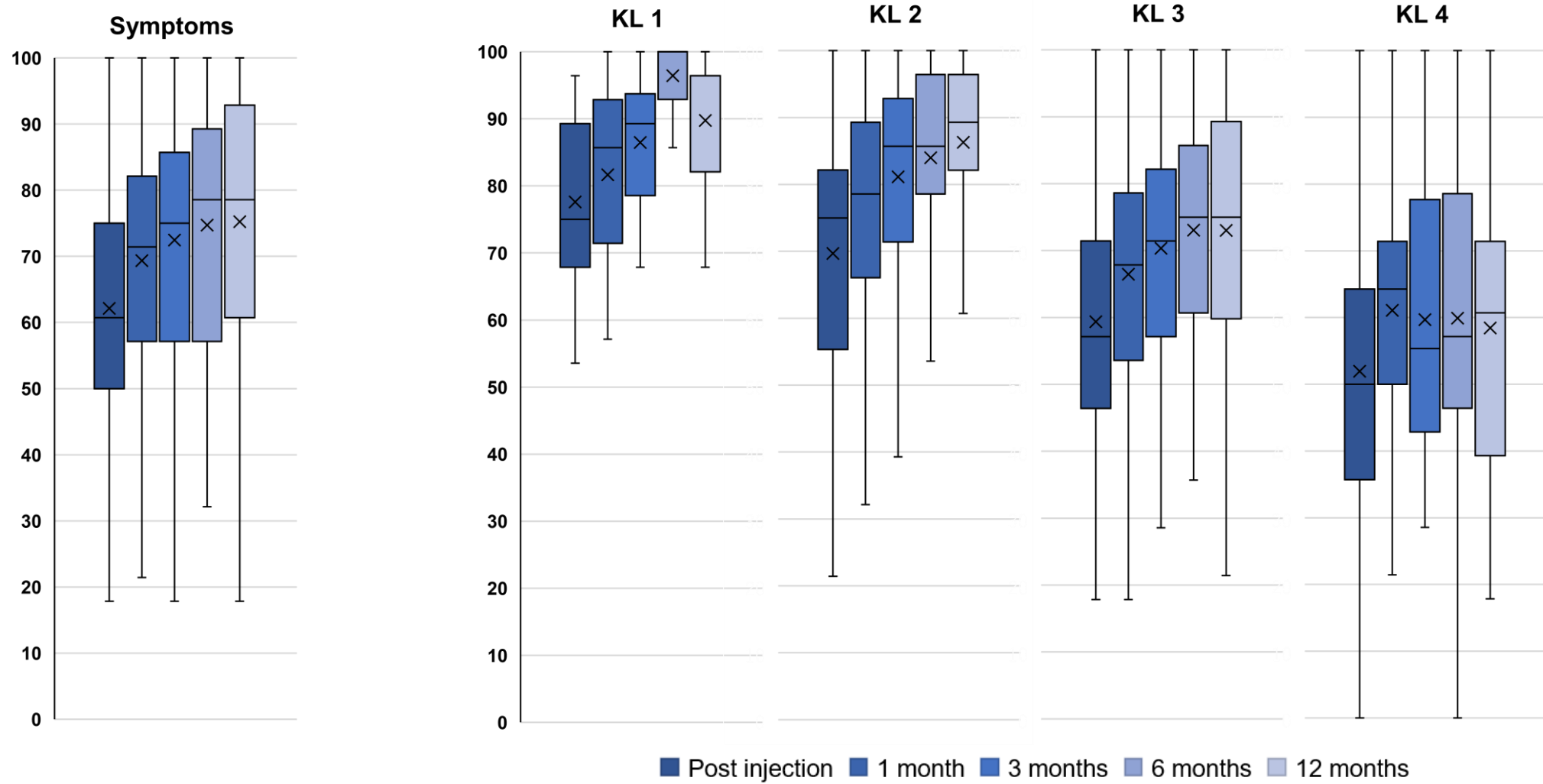

Supplement: Supplementary file 2 — Supplementary file2 (PDF 121 KB) [file 167_2023_7414_MOESM2_ESM.pdf]

**Supplementary Figure 5. KOOS Score change by grade of Knee Osteoarthritis. (Quality of Life)**

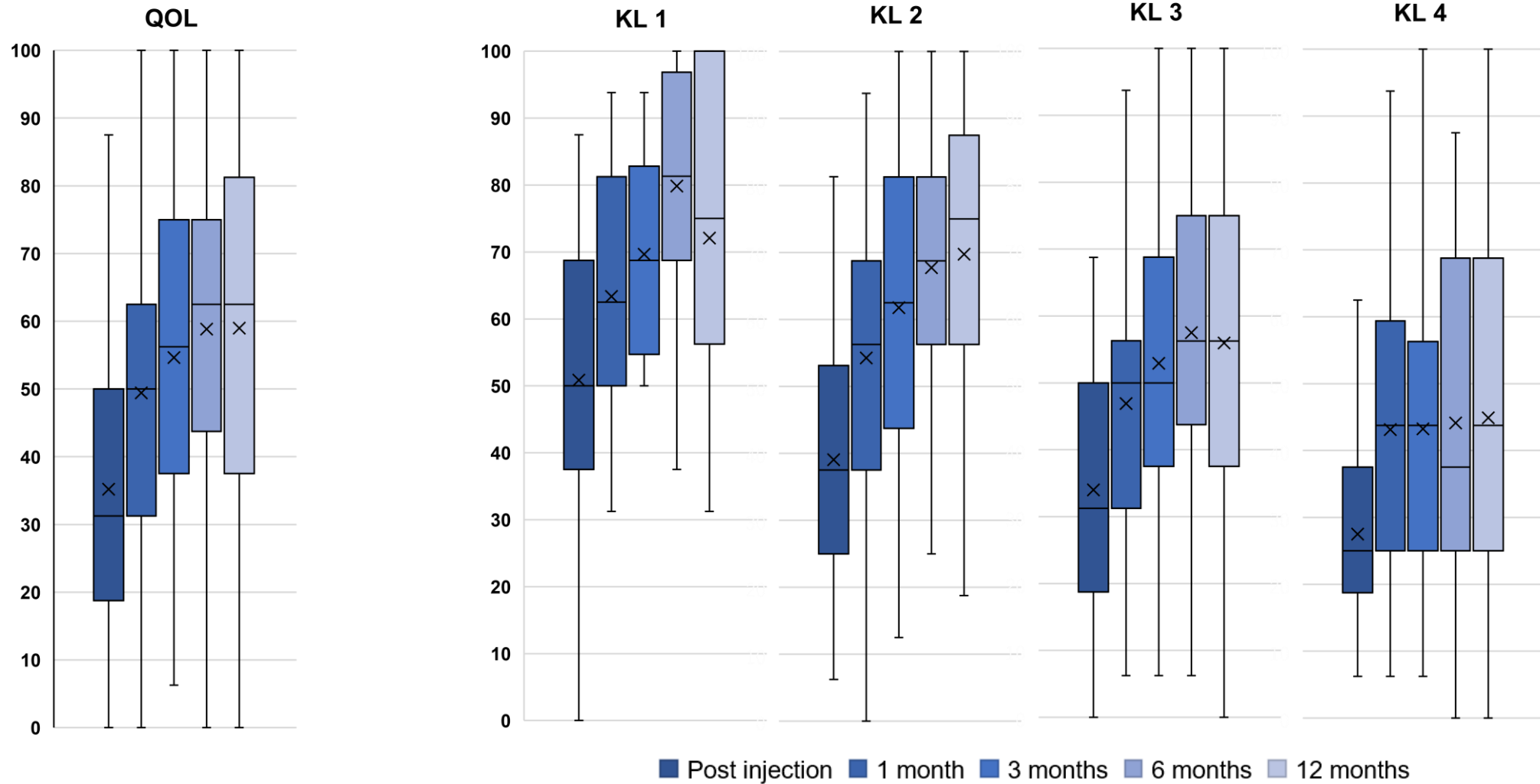

Supplement: Supplementary file 5 — Supplementary file5 (PDF 126 KB) [file 167_2023_7414_MOESM5_ESM.pdf]
