# Supplementary material for: Freeze-dried noncoagulating platelet-derived factor concentrate is a safe and effective treatment for early knee osteoarthritis
Source: Knee Surg Sports Traumatol Arthrosc. 2023 Jun 28;31(11):4716–23. doi: 10.1007/s00167-023-07414-y (PMC10598078; doi:10.1007/s00167-023-07414-y)

Supplementary Figure 3. KOOS Score change by grade of Knee Osteoarthritis. (Activities of Daily Living)

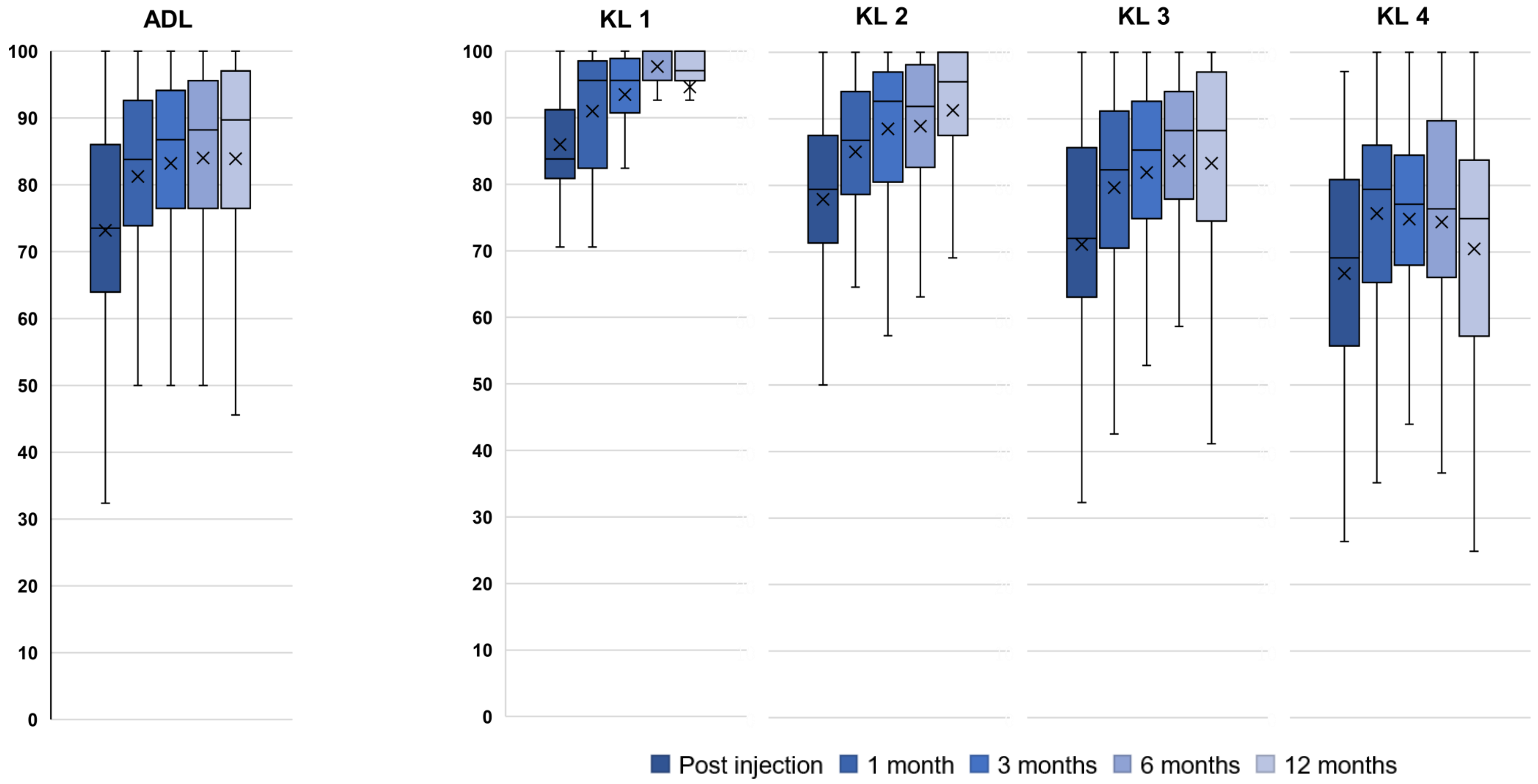

Supplement: Supplementary file 3 — Supplementary file3 (PDF 112 KB) [file 167_2023_7414_MOESM3_ESM.pdf]
