# Supplementary material for: Freeze-dried noncoagulating platelet-derived factor concentrate is a safe and effective treatment for early knee osteoarthritis
Source: Knee Surg Sports Traumatol Arthrosc. 2023 Jun 28;31(11):4716–23. doi: 10.1007/s00167-023-07414-y (PMC10598078; doi:10.1007/s00167-023-07414-y)

**Supplementary Table 1. PROMs scores at Baseline, 12 months, and the Delta (change from baseline to 12 months)**


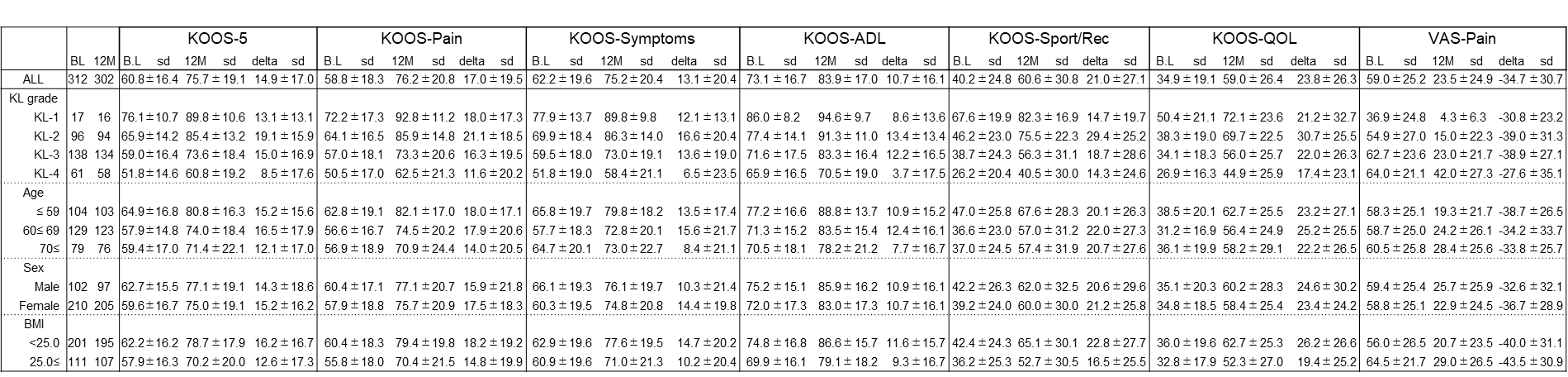

Supplement: Supplementary file 6 — Supplementary file6 (DOCX 60 KB) [file 167_2023_7414_MOESM6_ESM.docx]
